# Anatomical and histological characterization of the filum terminale in dogs

**DOI:** 10.3389/fvets.2025.1650893

**Published:** 2025-07-24

**Authors:** Christoforos Posporis, Javier Espinosa, Martí Pumarola, Ester Blasco Ortega, Jaume Alomar, Koen Santifort, Steven De Decker, Karol Lam, Juan José Mínguez, Patricia Álvarez, Vicente Aige-Gil

**Affiliations:** ^1^Neurology and Neurosurgery Department, Independent Vetcare (IVC) Evidensia, Pride Veterinary Referrals, Derby, United Kingdom; ^2^Mouse and Comparative Pathology Unit, Department of Animal Medicine and Surgery, Veterinary Faculty, Universitat Autònoma de Barcelona, Barcelona, Spain; ^3^Neurology and Neurosurgery Department, Independent Vetcare (IVC) Evidensia, Dierenziekenhuis Arnhem, Arnhem, Netherlands; ^4^Neurology and Neurosurgery Department, Independent Vetcare (IVC) Evidensia, Dierenziekenhuis Hart van Brabant, Waalwijk, Netherlands; ^5^Department of Clinical Science and Services, Royal Veterinary College, Hatfield, United Kingdom; ^6^Department of Animal Health and Anatomy, Veterinary Faculty, Universitat Autònoma de Barcelona, Barcelona, Spain

**Keywords:** dural sac, conus medullaris, ependyma, neurons, glia, collagen, tethered cord

## Abstract

The filum terminale (FT) remains poorly characterized in the veterinary literature, limiting understanding of its role in spinal cord and nerve root pathologies such as tethered cord syndrome. This study aimed to establish baseline anatomical and histological features of the FT in neurologically normal dogs. Eight adult canine cadavers euthanized for non-neurological reasons were examined. Dissection was performed via dorsal laminectomy (*n* = 4) and midline sectioning (*n* = 4). Histological analysis (*n* = 4) included hematoxylin and eosin, Klüver-Barrera, Masson’s trichrome, and Verhoeff-Van Gieson stains, alongside immunohistochemistry for neuron-specific enolase, protein gene product 9.5, and glial fibrillary acidic protein. Grossly, the FT was identified as a direct continuation of the conus medullaris within the region of the vertebral canal at L6, extending caudally to form two segments: a cranial portion within the subarachnoid space of the dural sac (filum terminale internum, FTi) and a caudal portion beyond the dural sac, enclosed by the inner dural layer without intervening subarachnoid space (filum terminale externum, FTe). The dural sac terminated at the level of mid-sacrum, with the FTe extending further caudally to insert dorsally between the first and second caudal vertebrae. The FT was accompanied by a ventral artery and vein. Histologically, the FT retained vestigial spinal cord cytoarchitecture, including an irregular, folded central canal with ependymal lining, a subependymal astrocytic layer, gray matter with scattered neurons and glial cells, and peripheral white matter with myelinated axons. The central canal and the neuronal and glial elements progressively diminished caudally as the FT transitioned to collagenous tissue interspersed with sparse residual nerve fibers. Verhoeff-Van Gieson staining revealed minimal elastic fibers within the collagen matrix. This study presents the first comprehensive anatomical and histological characterization of the normal canine FT, establishing baseline reference data to facilitate the identification of pathomorphological alterations associated with disorders involving this structure, such as tethered cord syndrome.

## Introduction

The filum terminale (FT) emerges during embryogenesis as a derivative of the neurulation process, contributing to the structural organization of the developing spinal cord. The embryological development of the neural tube involves both primary and secondary neurulation, with the latter giving rise to the conus medullaris (CM), ventriculus terminalis, cauda equina, and the FT (1–4). After closure of the caudal neuropore, a population of pluripotent cells—known as the caudal cell mass or tail bud—undergoes a gradual process of vacuolization, canalization, fusion with the cranial segment formed by primary neurulation, and retrogressive differentiation. This cascade of events plays a pivotal role in the formation of the FT (3–6). As the vertebral column and the external layer of the dura mater grow at a rate that surpasses that of the spinal cord during fetal development, the CM ascends within the vertebral canal, contributing to the extension of the FT from its original caudal attachment (1).

The gross anatomy of the FT remains poorly documented in dogs (7). In humans, the FT is a slender, fibro-neural structure that extends caudally from the apex of the CM to the coccygeal vertebrae within the vertebral canal. It is divided into cranial and caudal portions (or fila) based on its meningeal coverage. The cranial portion, also known as the FT internum (FTi), is surrounded by the leptomeninges and the inner layer of the dura mater, which contributes to forming the dura-arachnoid sheath known as the dural sac (DS). The DS extends to the sacral vertebrae and contains cerebrospinal fluid (CSF), spinal nerve roots, and blood vessels within the subarachnoid space. Immediately caudal to the DS, the inner layer of the dura mater constricts and envelops the caudal portion of the FT without intervening subarachnoid space. This segment of the FT is referred to as the FT externum (FTe) and extends until its attachment on the coccygeal vertebrae (8, 9).

The functional properties and histological characteristics of the FT have been extensively documented in humans (8–13) but similar descriptions are lacking in dogs. Histologically, it consists of type I collagen fibers arranged longitudinally and type III collagen fibers oriented transversely, with abundant elastic and elaunin fibers contributing to its elasticity (10). Nervous tissue, including neurons, nerve fibers, glial cells, and ependymal cells, is present and is often regarded as a remnant of secondary neurulation. However, the presence of mechanoreceptors and nociceptors (14) suggests functional involvement in sensory pathways, while motor responses elicited during FT electrical stimulation (15–17) indicate preservation of motor pathway elements. In contrast, data on the canine FT remain limited (18, 19) and are predominantly described in the context of pathological conditions (5, 20–22), similar to findings in other species (23).

Filum terminale pathology is well characterized in humans (24, 25) but remains poorly understood and infrequently reported in animals (5, 21). Tethered cord syndrome (TCS) is attributed to a shortened, thickened, or inelastic FT, resulting in pathological tension on the spinal cord and progressive neurological dysfunction (26–28). In dogs, TCS is rarely diagnosed and typically occurs alongside congenital anomalies, including spina bifida and/or meningocele/meningomyelocele (2, 29, 30). Occult TCS, characterized by signs of cord tethering despite normal conventional MRI findings, is even less common and poses significant diagnostic challenges (22, 31, 32). The underlying etiopathogenic contribution of the FT to these conditions remains uncertain, partly due to the lack of detailed anatomical and histological data in healthy dogs. This absence of normative reference standards limits the ability to recognize pathological deviations, impeding diagnostic accuracy and mechanistic understanding.

This study aims to describe the anatomical and histological features of the FT in neurologically normal dogs. Establishing these baseline characteristics provides a critical foundation for recognizing disease-associated alterations and may ultimately enhance diagnostic accuracy for tethered cord syndrome in veterinary patients, particularly in its occult form, where conventional MRI fails to reveal abnormalities. These findings may also contribute to more accurate post-surgical histopathological diagnostic confirmation and a better understanding of disease mechanisms in veterinary patients.

## Materials and methods

Eight adult dogs of variable body weights and breeds (Supplementary Table 1), euthanized for medical reasons unrelated to the central nervous system, were included in this study. The animals were donated by their owners through the approved deceased animal donation program at the Universitat Autònoma de Barcelona (UAB). The dogs were randomly selected from the donation program, with exclusion criteria consisting of any history or evidence of neurological or orthopedic disease that might affect the normal anatomy or histology of the FT and surrounding structures. All procedures were conducted in accordance with the applicable national and institutional guidelines for the use of animals in research, and informed consent was obtained from all owners prior to the donation of the cadavers. These specimens were used strictly for anatomical education and research purposes. The cadavers were preserved using a 10% formaldehyde solution (200 mL/kg) injected within 24 h after euthanasia through the common carotid artery and subsequently stored at a temperature of 4–6°C for several weeks.

Cadaver dissection of the lumbar, sacral, and caudal vertebral column and associated structures was performed. A dorsal laminectomy was conducted in one Beagle, one Labrador Retriever, one Golden Retriever, and one German Shepherd to expose the spinal cord and FT. Additionally, one Border Collie and three middle-size mixed-breed dogs were sectioned along the mid-sagittal plane after being frozen at −20°C to facilitate precise division using a saw. While frozen specimens were not suitable for histological analysis, they provided high-resolution anatomical images, allowing for detailed visualization of the FT and its terminal attachment. Gross anatomical morphologic analysis focused on characterizing the filum terminale and determining its spatial relationships with surrounding structures, with anatomical positions defined in relation to whole vertebral body segments.

Histological samples were collected from the four specimens that had not been frozen. The FT from the first three specimens was sectioned longitudinally, while the FT from the fourth specimen was sectioned transversely at multiple vertebral levels, including the lumbosacral junction, first sacral vertebra (S1), second to third sacral vertebrae (S2–S3), and first to second caudal vertebrae (Cd1–Cd2). All samples were fixed in 10% buffered formalin, processed using standard histological techniques, and stained with various methods: hematoxylin and eosin (H&E) for general tissue structure, Klüver-Barrera (KB) for myelin sheaths and Nissl granules, Masson’s trichrome for collagen fibers, and Verhoeff-Van Gieson (VVG) for elastic tissue.

Additional slides were processed for immunohistochemical (IHC) analysis to detect neuron-specific enolase (NSE) and protein gene product 9.5 (PGP 9.5) as markers for neural cell bodies and axons, and glial fibrillary acidic protein (GFAP) as a marker for astrocytes. The IHC was performed using a biotin-peroxidase detection system, with diaminobenzidine (DAB) as the chromogenic substrate. Sections (3 μm thick) were mounted on adherent glass slides, deparaffinized, and rehydrated. Endogenous peroxidase activity was quenched by incubation with 3% hydrogen peroxide for 35 min at room temperature (RT). Antigen retrieval was performed using 0.01 M citrate buffer (pH 6.0) in a pressure cooker. Nonspecific binding was blocked by incubation with 30% normal goat serum diluted in phosphate-buffered saline (PBS) for 1 h at RT. Sections were then incubated overnight at 4°C with the following primary antibodies: mouse monoclonal anti-NSE (1:600 dilution; Dako, Denmark), rabbit polyclonal anti-PGP 9.5 (1:1800 dilution; Cedarlane Labs, Canada), and rabbit polyclonal anti-GFAP (1:5000 dilution; Dako, Denmark). After washing with PBS, sections were incubated for 40 min at RT with horseradish peroxidase-conjugated secondary antibodies according to the manufacturer’s protocol (NeoStain Poly 1-Step Kit, Horseradish Peroxidase Detection System for Rabbit or Mouse Antibodies; Neo Biotech, France). Immunoreactivity was visualized by incubation with DAB substrate for 10 min. Sections were counterstained with hematoxylin for 3 s, dehydrated, and cover-slipped. For negative controls, primary antibodies were replaced with isotype-matched immunoglobulins at equivalent concentrations; no immunoreactivity was observed in these sections.

## Results

### Macroscopic anatomy

**Figure 1:**
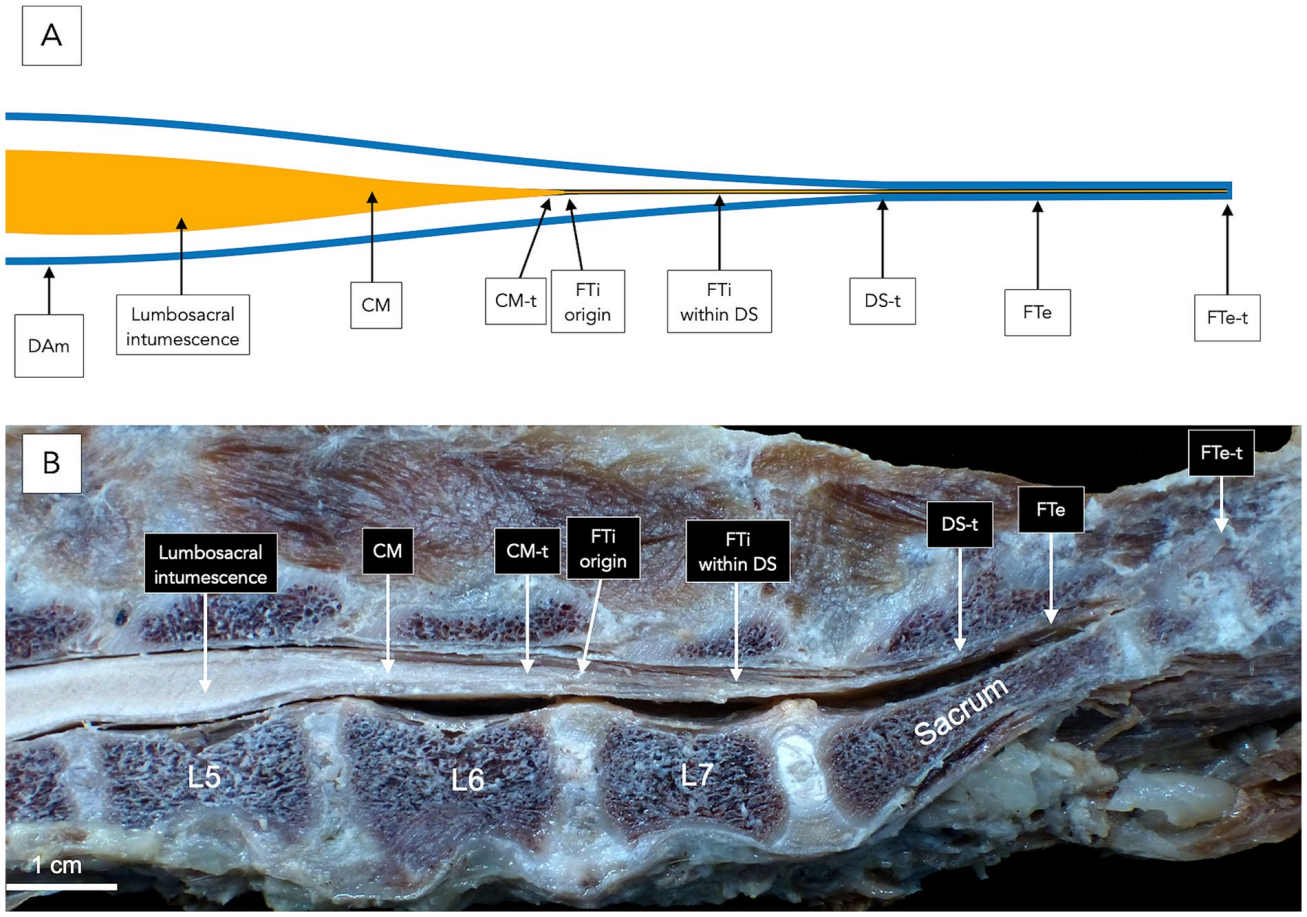
Anatomical illustration **(A)** and midline gross specimen **(B)** of the filum terminale (FT) and associated structures in a dog. Both panels depict the conus medullaris (CM), FT, and surrounding anatomy at the caudal lumbar, sacral, and first two caudal vertebral levels. The lumbosacral intumescence tapers into the CM, the termination of which (CM-t) marks the origin of the FT. The FT internum (FTi) extends from the CM-t to the dural sac termination (DS-t) and is surrounded by cerebrospinal fluid within the subarachnoid space, enclosed by the dura mater and arachnoid mater (DAm), collectively known as the dural sac (DS). The filum terminale externum (FTe) continues caudally beyond the DS-t, where it is ensheathed by the inner dura mater without intervening cerebrospinal fluid or subarachnoid space. The FTe termination (FTe-t) anchors to the interarcuate space at the ligamentum flavum between the first and second caudal vertebrae (Cd1–Cd2).

The anatomical configuration was largely consistent among the examined cadaveric specimens, with any positional variations confined within the same vertebral segment. A schematic illustration of the macroscopic anatomy of the CM, FT, and associated structures is provided in Figure 1A, alongside a mid-sagittal section of a formaldehyde-preserved cadaveric canine specimen encompassing the caudal lumbar, sacral, and first two caudal vertebral regions (Figure 1B).

**Figure 2:**
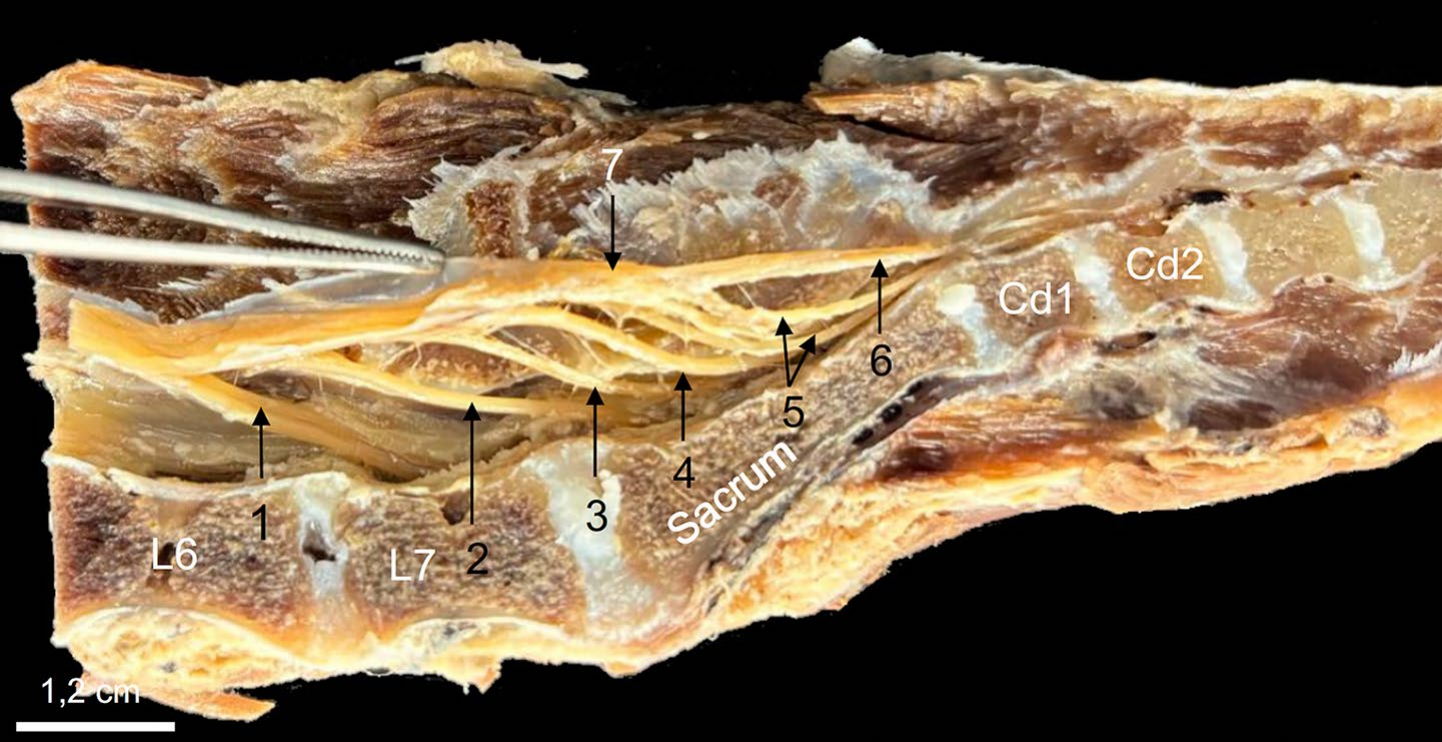
Midline sagittal section of a formaldehyde-preserved specimen showing the caudal lumbar, sacral, and first four caudal vertebrae, demonstrating the anatomical components of the cauda equina. The L7 (1), S1-S3 (2, 3, 4), and first two caudal (5) spinal nerve roots course caudally from their origin in the lumbosacral intumescence and conus medullaris to form the cauda equina. The dural sac has been opened, and the dura mater is retracted upwards using forceps to expose the spinal nerve roots of interest. The filum terminale externum (6) is identified in direct caudal extension of the dural sac (7).

The lumbosacral intumescence was identified at the level of the fifth lumbar vertebra (L5). Caudal to this, the spinal cord tapered progressively, forming the CM at the level of the sixth lumbar vertebra (L6). The CM terminated within the region of L6 (Figure 1). The spinal nerve roots originating from the CM extended caudally to constitute the cauda equina. The cauda equina occupied the lumbo-sacral and caudal vertebral canal regions and comprised the seventh lumbar (L7), sacral (S1-S3), and caudal spinal nerve roots (Figure 2).

**Figure 3:**
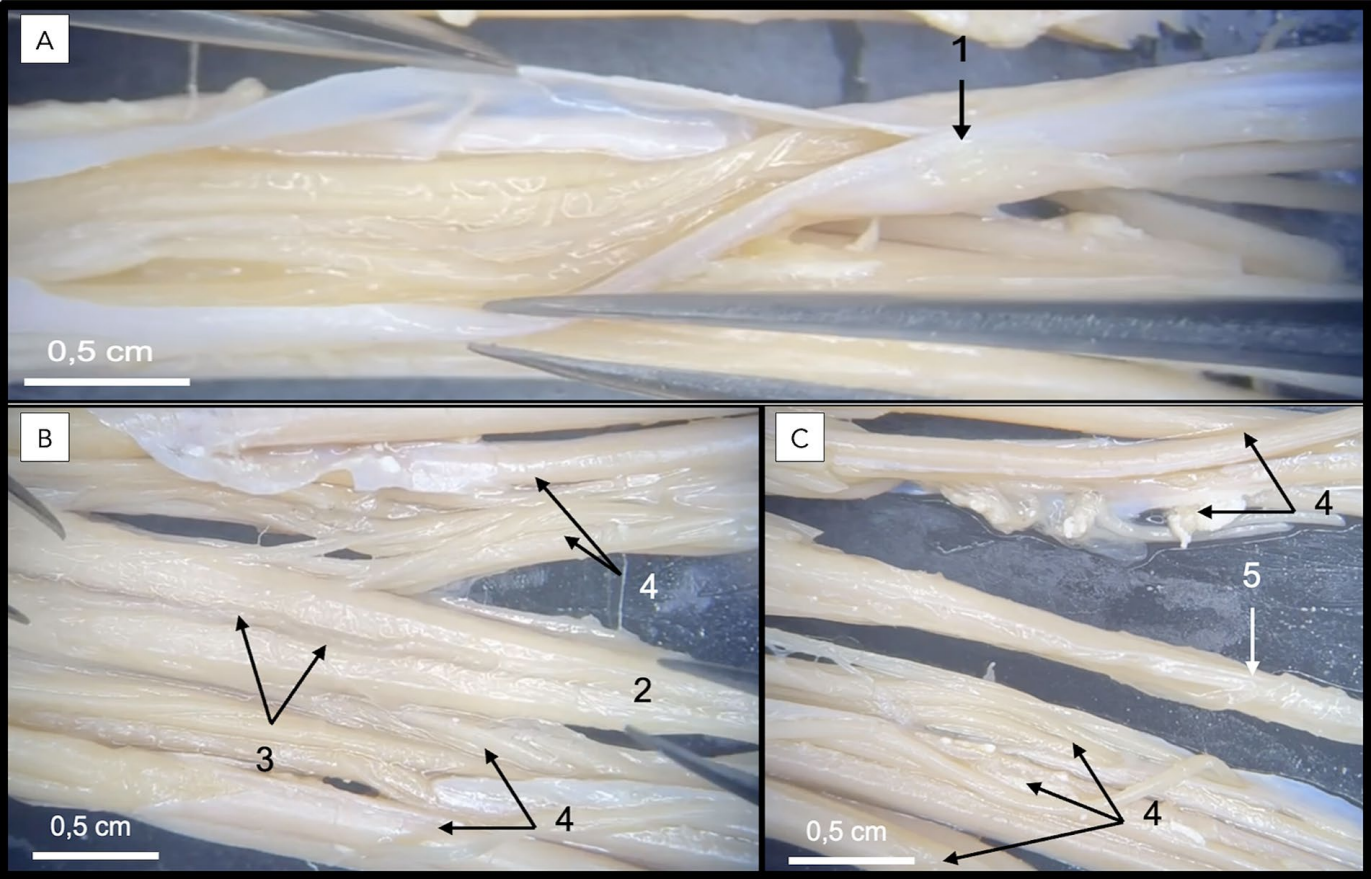
Dissection of a formaldehyde-preserved canine specimen demonstrating a ventral view of the dural sac and filum terminale. In **A**, the dural sac is observed following incision of the inner (meningeal) layer of the dura mater (1), exposing the intradural components. In **B**, complete removal of the dura mater provides an unobstructed view of the filum terminale internum (2) coursing centrally along the midline, closely associated with the ventral spinal artery (3) and spinal nerve roots (4). In **C**, the filum terminale externum (5), representing the continuation of the filum terminale internum beyond the dural sac termination, is also identified.

**Figure 4:**
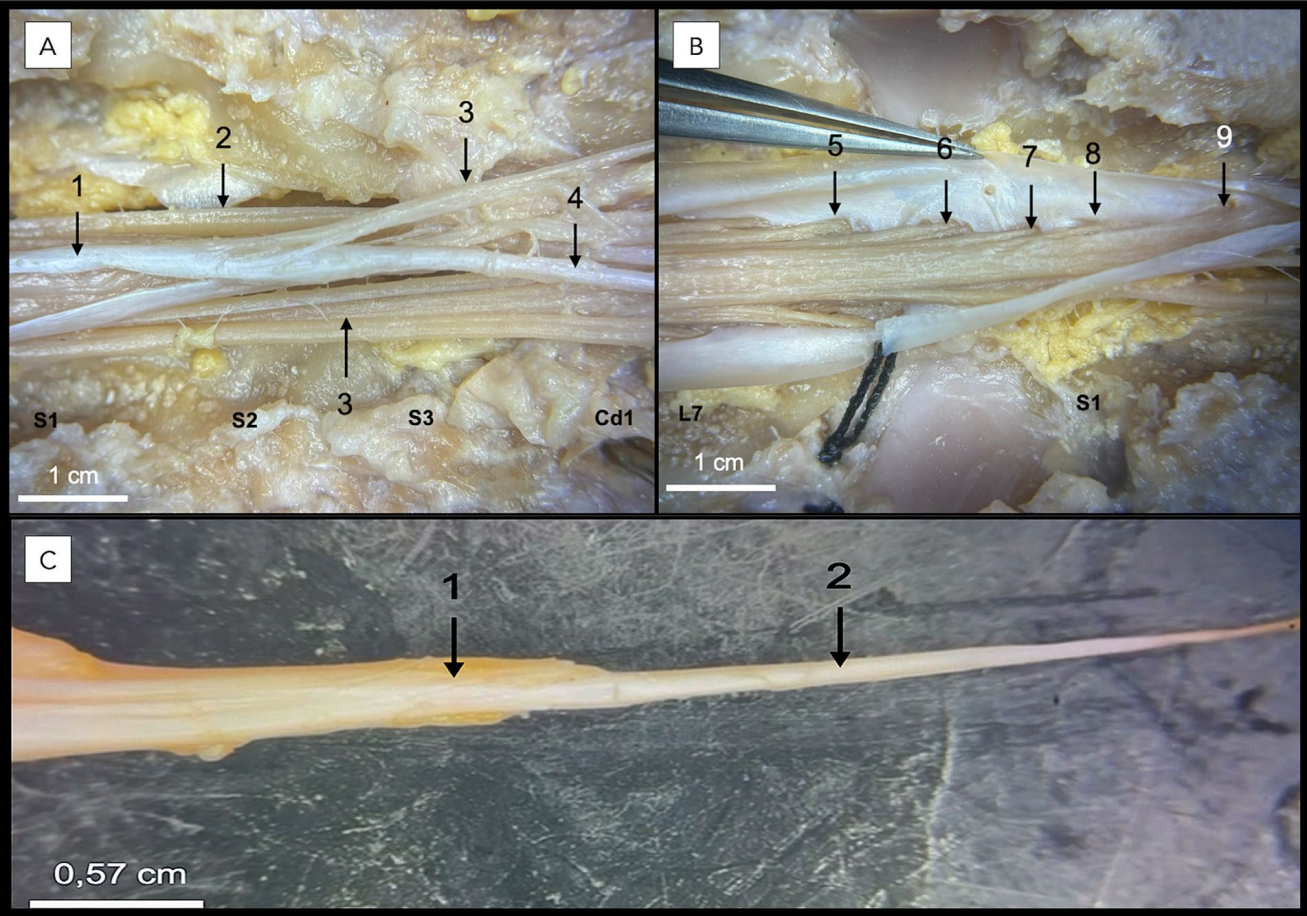
Dissection following a lumbosacral dorsal laminectomy of a formaldehyde-embalmed canine cadaveric specimen. In **A,B**, the seventh lumbar (L7), three sacral (S1, S2, S3), and first caudal (Cd1) vertebrae are identified. The dura mater (1) of the opened dural sac, sacral spinal nerve roots (2), caudal spinal nerve roots (3), and filum terminale externum (4) are visible in **A**. In **B**, the S1 (5), S2 (6), S3 (7), Cd1 (8), and Cd2 (9) spinal nerve roots are identified within the dural sac. In **C**, the filum terminale externum (2) is seen as a direct caudal continuation of the dural sac termination (1).

**Figure 5:**
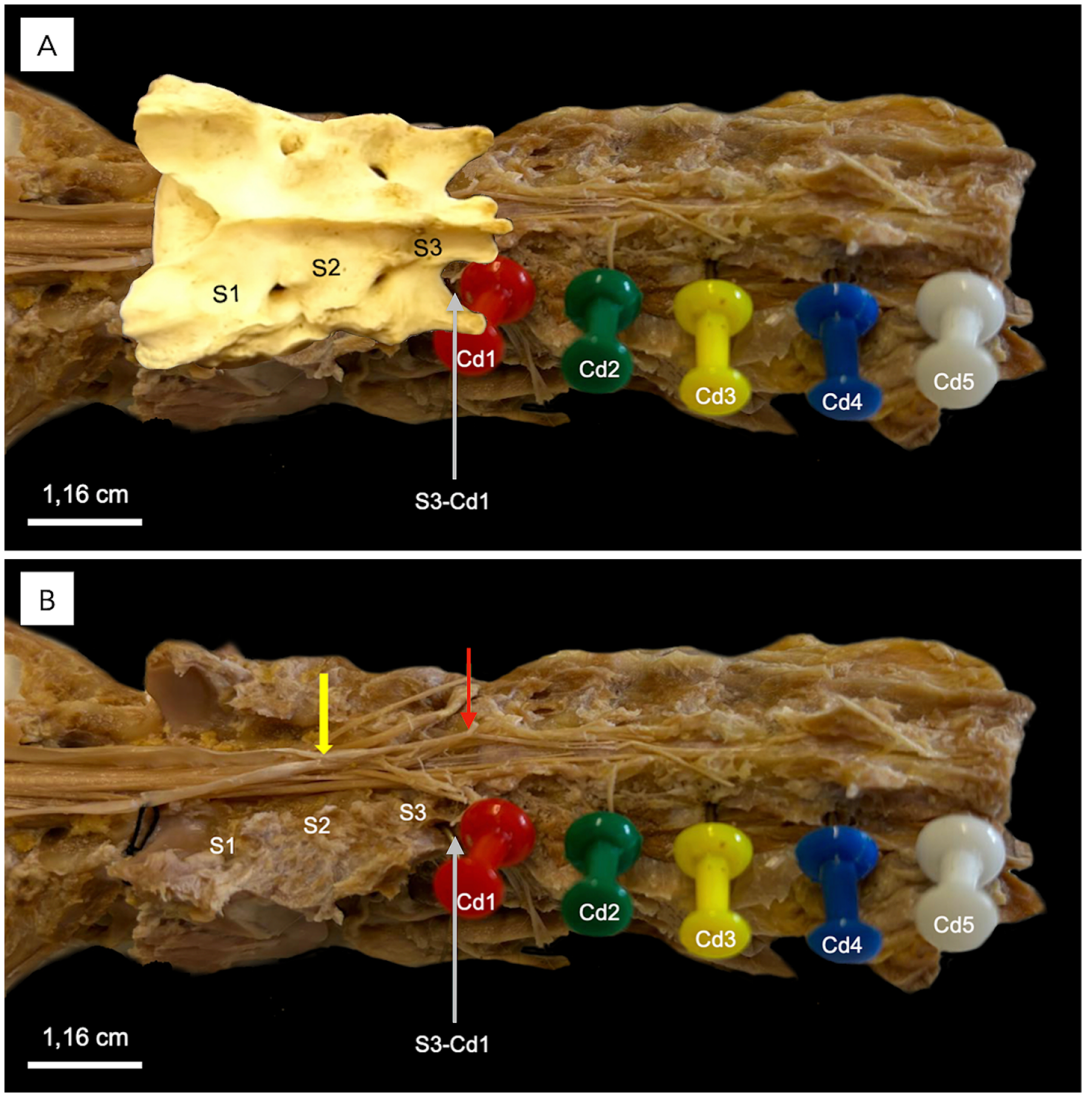
Dorsolateral view of the sacral and caudal vertebrae of a middle-size dog following a complete dorsal laminectomy. In **A**, colored needles indicate the caudal vertebrae (Cd1–Cd5). The sacral bone is overlaid in this image, with S1, S2, and S3 marking the sacral vertebrae. In **B**, the sacral bone has been removed from the image. The yellow arrow points to the termination of the dural sac at the S2-S3 vertebra, while the red arrow highlights the filum terminale externum and its dissected dorsal attachment to Cd1-Cd2 interarcuate space and ligamentum flavum.

**Figure 6:**
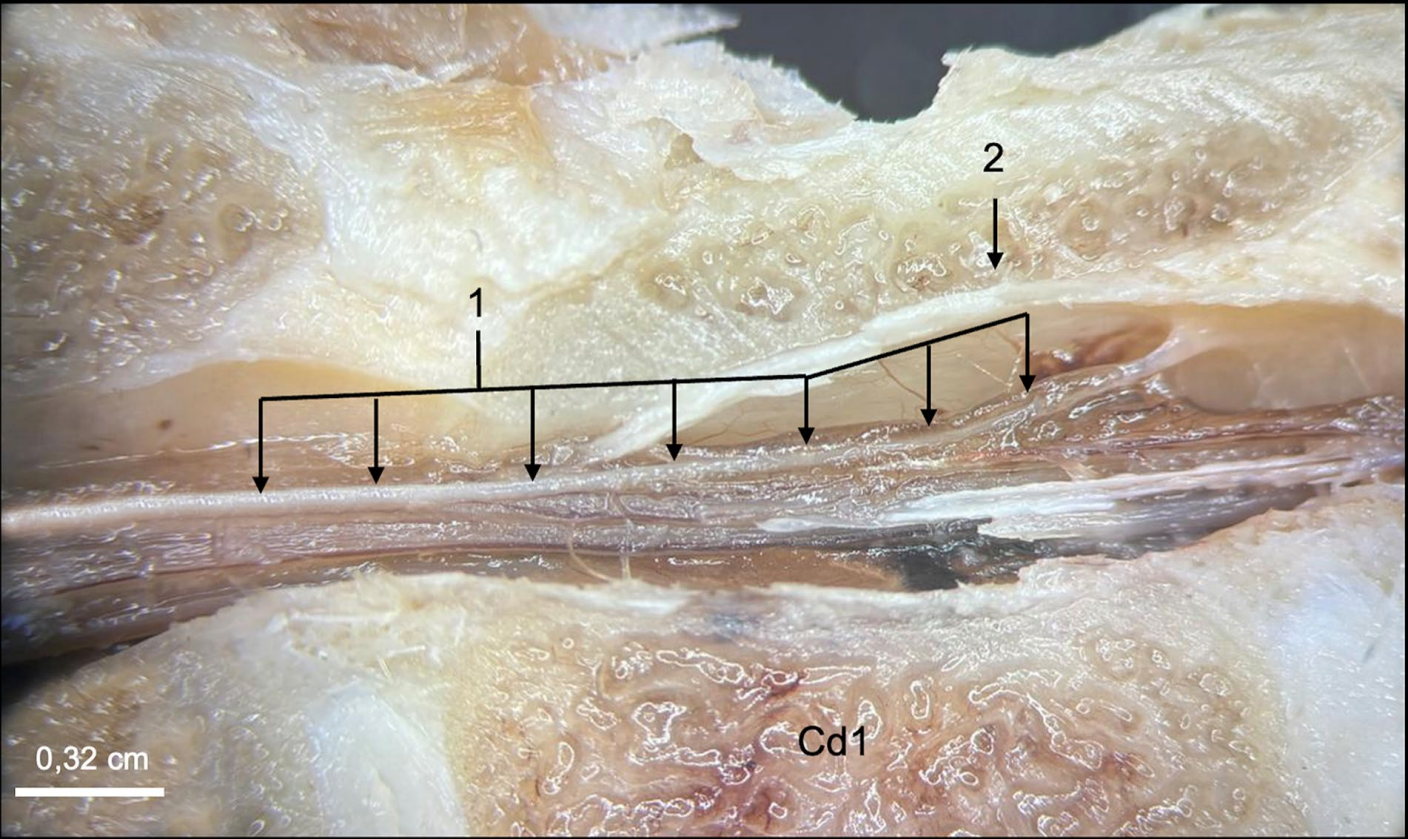
Close-up view of the filum terminale externum at the level of the first caudal vertebra (Cd1) in a midline plane section. The filum terminale externum (1) and its dorsal attachment to the vertebral canal (2), corresponding to the coccygeal ligament in human anatomy, are visualized.

In direct anatomical continuity with the apex of the CM, the origin of the FT was identified at the L6–L7 intervertebral disk space (Figure 1). The cranial segment of the FT, herein defined as the FTi, was observed within the subarachnoid space of the DS as it coursed caudally through the L7 and first two sacral vertebrae, extending from the termination of the CM to the termination of the DS (Figure 1). The FTi appeared as a delicate, slender, pale filament, situated in a dorsal midline position within the DS, and displayed a smooth, uniform appearance. Closely associated with the ventral surface of the FTi, the ventral artery and vein of the FT were consistently observed as direct continuations of the ventral spinal artery and vein (Figure 3). These vessels coursed along the ventral length of the FT, providing its principal arterial supply and venous drainage, respectively. The DS consisted of the arachnoid membrane and the meningeal (inner) layer of the dura mater, while the outer periosteal layer was closely adherent to the vertebral canal. The DS extended caudally and terminated at the level of the second to third sacral vertebrae (mid-sacrum; S2-S3; Figures 1, 4, 5). Beyond this point, the FT was no longer accompanied by the subarachnoid space but remained directly ensheathed by the inner layer of the dura mater—also known as the *filum durae matris spinalis* or dural filament (Figures 1, 4, 5). This caudal segment, referred to as the FTe, spanned from mid-sacrum to its point of dorsal insertion at the *ligamentum flavum* (interarcuate ligament), located between the vertebral arches of the first two caudal vertebrae (Cd1–Cd2) as shown in Figures 1, 6. The FTe appeared as a fibrous, tapering band. Its surface was smooth but distinctly more robust compared to the softer, more delicate appearance of the FTi (Figures 4, 6).

### Histological findings

Histological examination of the four cadaveric specimens revealed consistent morphological features of the FT, enabling detailed characterization of its cytoarchitecture and cellular composition. The findings described below were consistent across all specimens, supporting a conserved histological organization of the canine FT.

**Figure 7:**
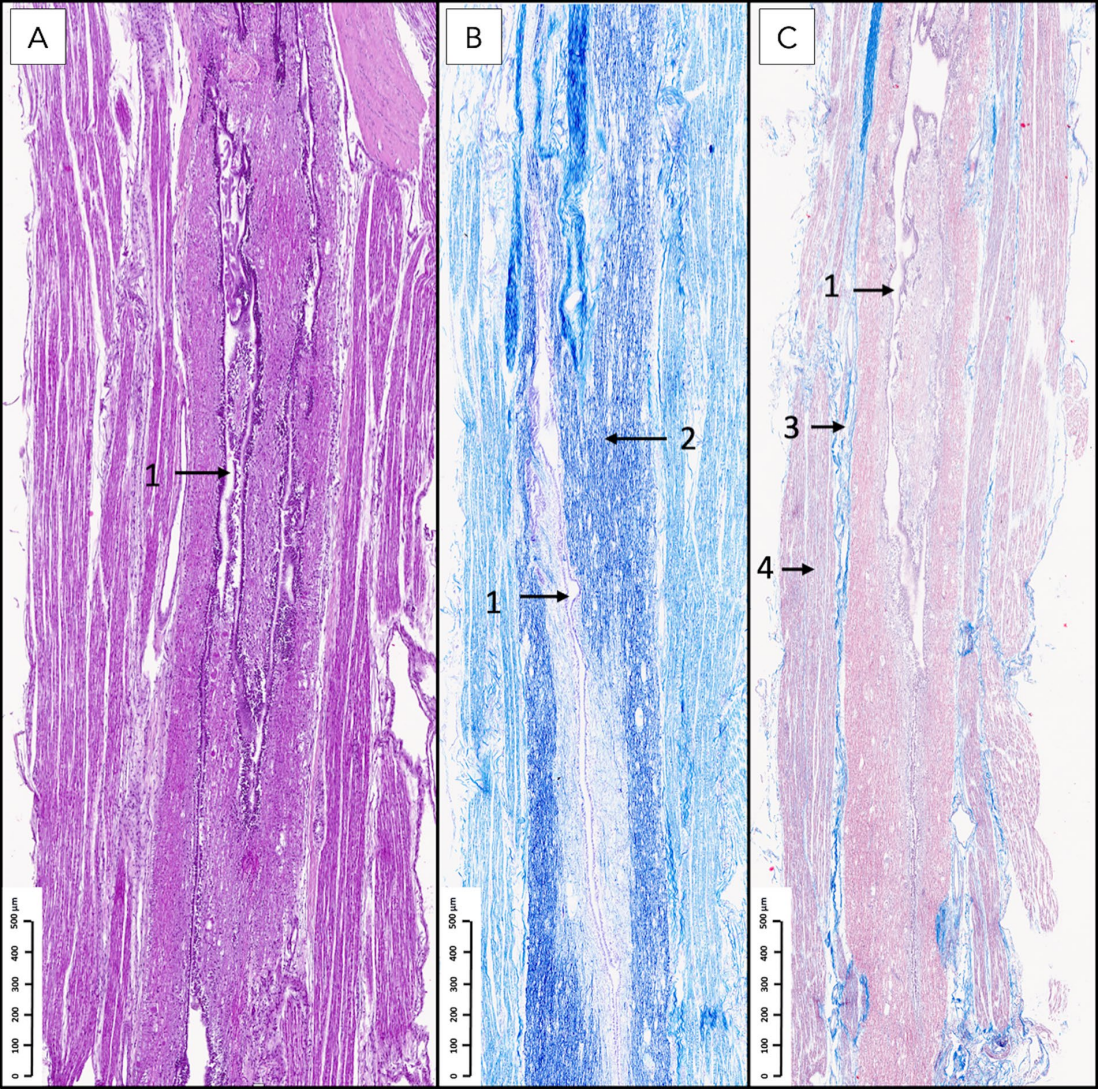
Histological sagittal sections of the filum terminale internum at the lumbosacral vertebral level stained with hematoxylin and eosin **(A)**, Kluver–Barrera **(B)**, and Masson’s trichrome **(C)**. A longitudinally oriented, irregular central canal (1) with occasional folds is observed. In panel **B**, intense blue staining corresponds to white matter myelin (2). The dura mater (3) surrounds the white matter, and spinal nerve roots (4) are visualized overlaying the dura, consistent with their anatomical relationship with the dural sac.

**Figure 8:**
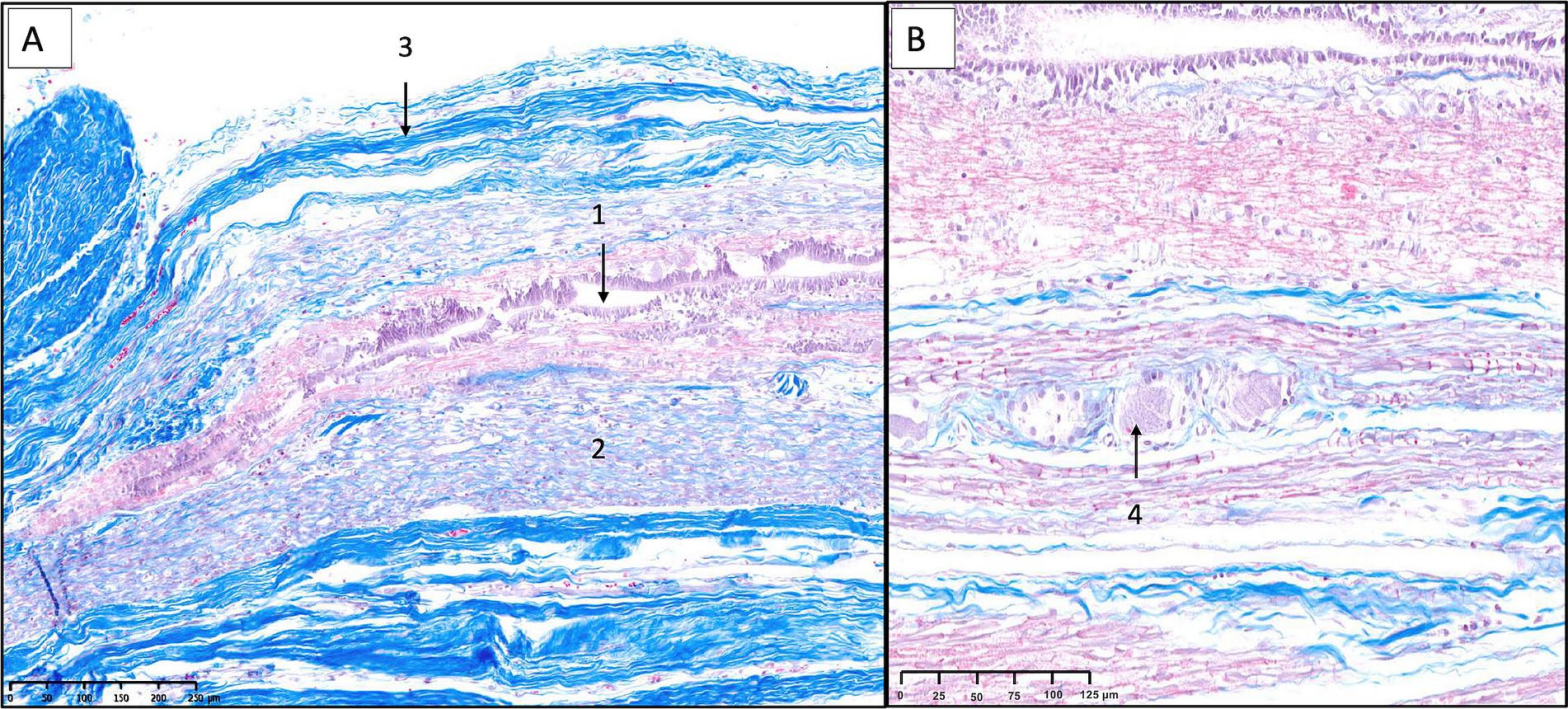
Sagittal histological sections of the filum terminale internum at the lumbosacral junction in a canine specimen, stained with Masson’s trichrome. In panel **A**, the central canal (1), lined by ependymal cells, is surrounded by residual neural tissue containing scattered glial cells and neurons. Longitudinally oriented axonal bundles (2) are embedded within endoneurium, while the outermost layer consists of dense, blue-stained collagenous connective tissue of dural origin (3). In panel **B**, at higher magnification, isolated ganglion cells (4) are visible within the surrounding endoneurial connective tissue, with blue-stained collagen fibers delineating the fibroconnective scaffold.

**Figure 9:**
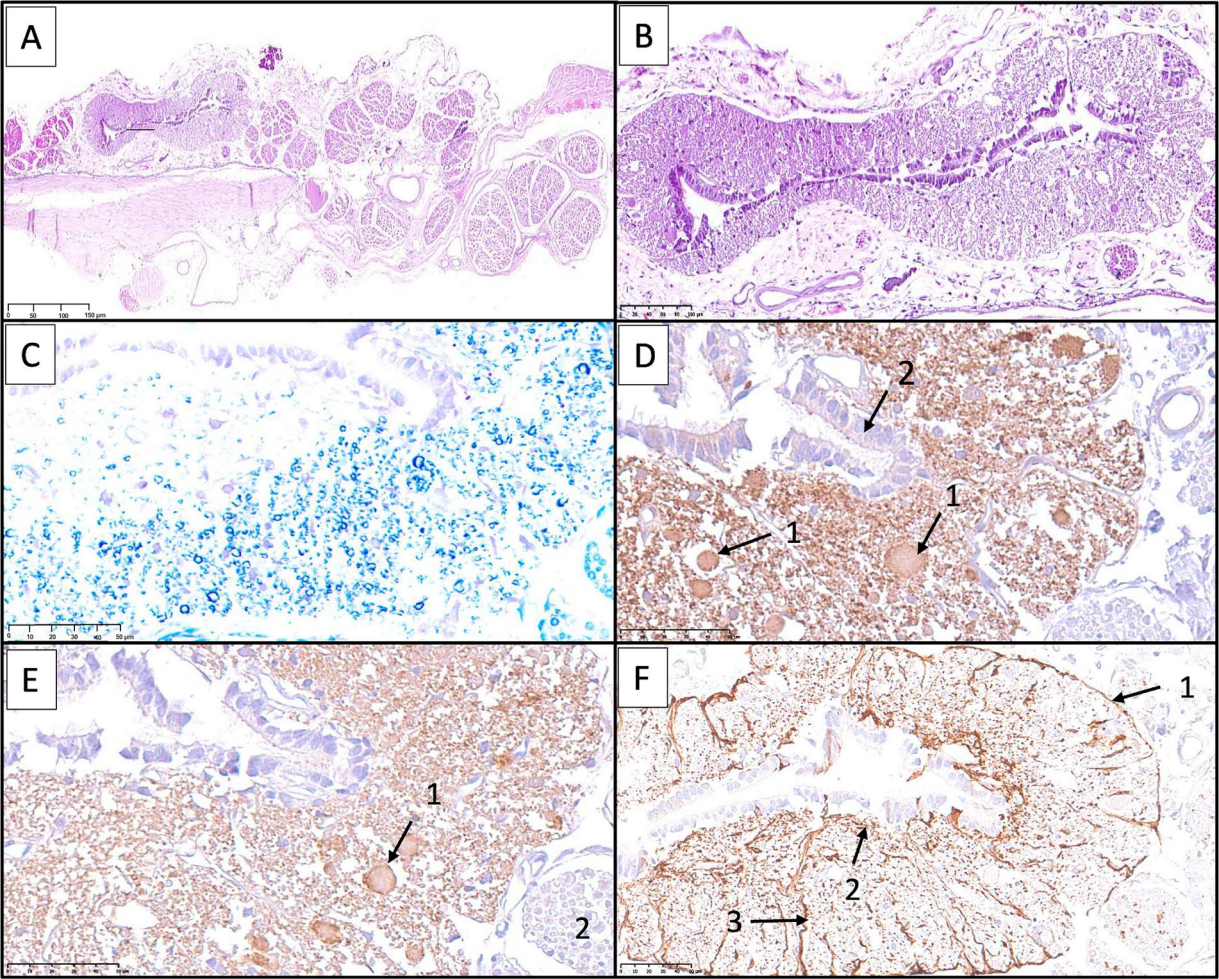
Histological and immunohistochemical examination of the filum terminale internum at the lumbosacral junction in a canine specimen. **(A)** Transverse section stained with hematoxylin and eosin (H&E) showing the residual spinal cord and surrounding nerve roots and connective tissues. **(B)** Higher magnification of the residual spinal cord showing central canal lined by ependymal cells, with adjacent nervous tissue containing scattered neurons and glial cells. **(C)** Kluver-Barrera staining highlights small neuronal bodies in the gray matter and numerous myelinated axons filling the adjacent white matter. **(D)** Immunostaining for neuron-specific enolase (NSE) reveals immunoreactivity in neurons of varying sizes (1). Ependymal cells (2) are also seen. **(E)** Immunostaining for protein gene product 9.5 (PGP 9.5) demonstrates robust labeling of neurons (1) and nerve fibers (2). **(F)** Immunostaining for glial fibrillary acidic protein (GFAP) shows a continuous astrocytic layer (glia limitans externa) adjacent to the pia mater (1), astrocytic processes attached to the ependymal layer (2), and perivascular astrocytic endfeet surrounding blood vessels (3).

**Figure 10:**
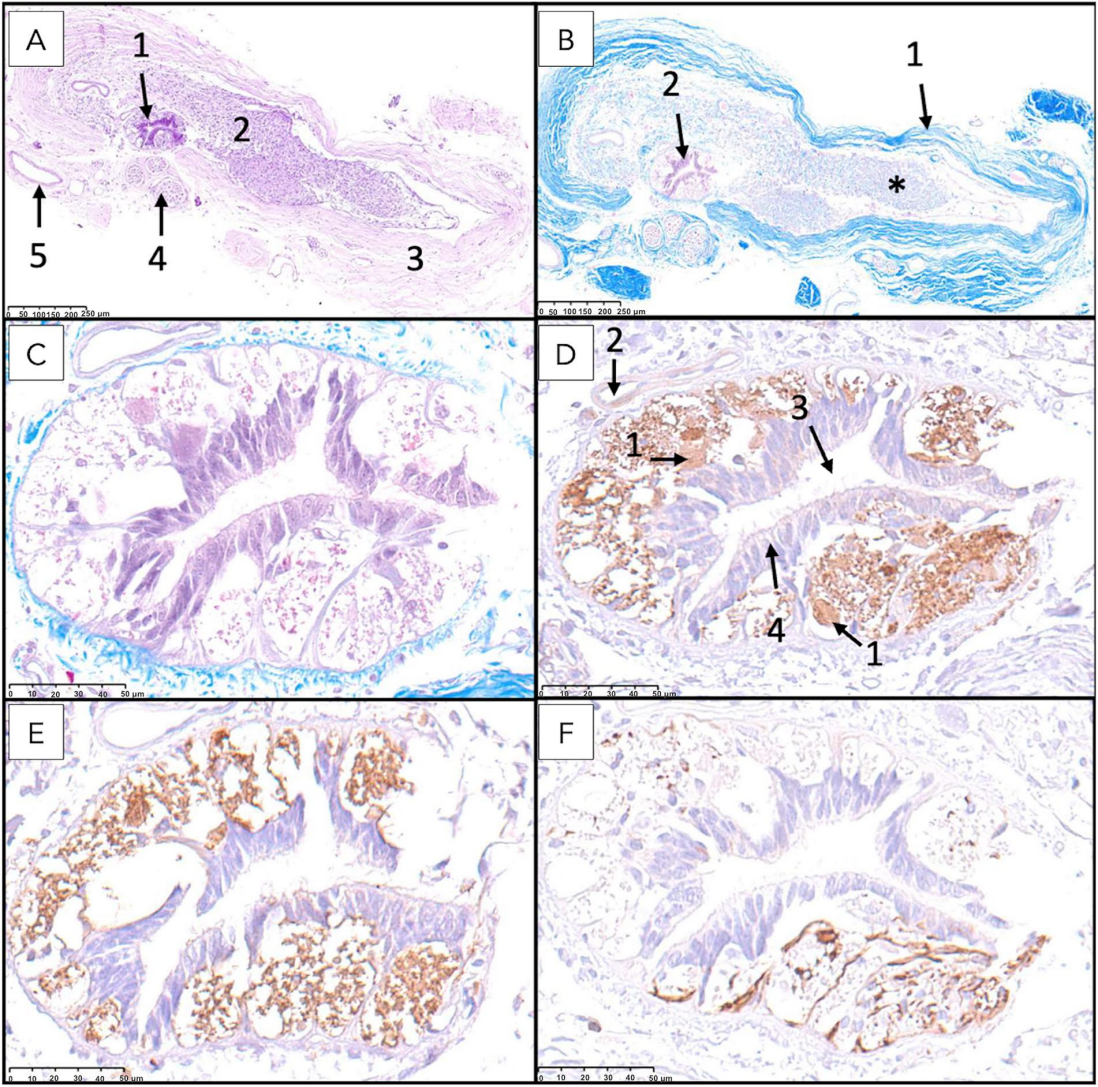
Histological and immunohistochemical analysis of the filum terminale internum at the first and second sacral vertebral level. **(A)** Transverse section stained with hematoxylin and eosin (H&E) demonstrates overall tissue architecture, with the central canal surrounded by neural tissue (1), intradural myelinated nerve fibers (2), dura mater (3), nerve roots (4), and blood vessels (5) visible. **(B)** Kluver-Barrera staining reveals myelinated fibers (*) in the filum terminale, with distinct dura mater (1) and central canal (2). **(C)** Higher magnification of Kluver-Barrera staining shows detailed organization of the central canal and surrounding nervous tissue. **(D)** Immunostaining for neuron-specific enolase (NSE) demonstrates immunoreactivity in neuronal cell bodies (1), with visible blood vessels (2), central canal (3), and ependymal cells (4). **(E)** Protein gene product 9.5 (PGP 9.5) immunostaining shows strong positivity in neuronal cell bodies. **(F)** Glial fibrillary acidic protein (GFAP) immunostaining reveals robust expression in astrocytes.

**Figure 11:**
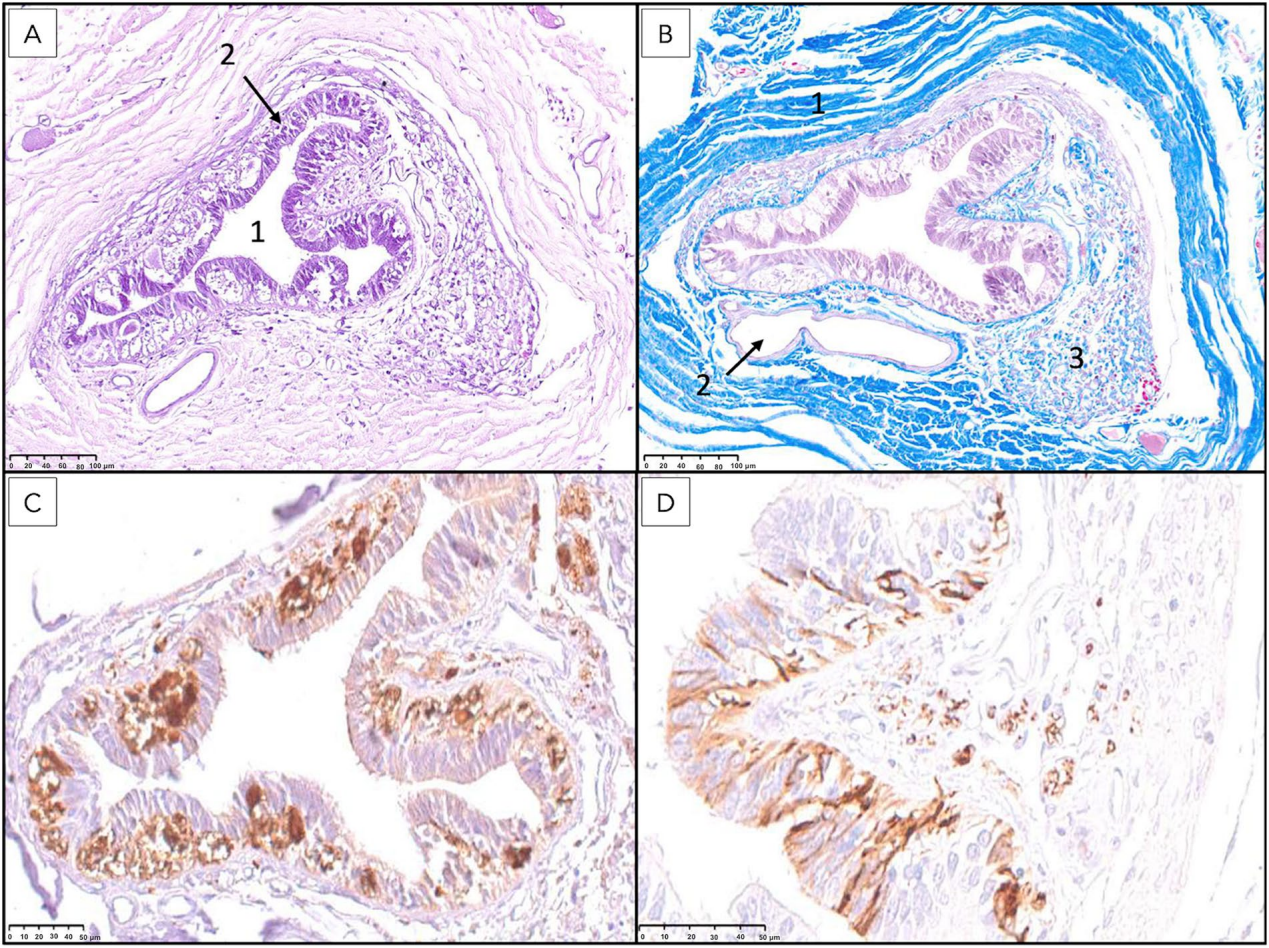
Histological and immunohistochemical analysis of the filum terminale externum at the third sacral vertebral level. **(A)** Hematoxylin and eosin staining reveals the central canal (1) lined by ependymal cells (2) and surrounded by neural tissue. **(B)** Masson’s trichrome staining highlights the dura mater (1), a prominent blood vessel corresponding to the ventral spinal artery (2), and regions of immature mesenchymal tissue (3). **(C)** Immunostaining for protein gene product 9.5 (PGP 9.5) demonstrates strong positivity in neuronal cell bodies. **(D)** Immunostaining for glial fibrillary acidic protein (GFAP) shows robust expression in astrocytes reaching with their processes the lumen of the canal passing between ependymal cells.

Sagittal sections of the FTi (Figures 7, 8) demonstrated a caudal extension of the spinal cord with vestigial cytoarchitecture. The central canal, lined with ependymal cells, was represented by multiple irregular elongated cavities observed in longitudinal sections that were determined to be folded segments of a single, irregularly contoured central canal, as corroborated by corresponding transverse sections (Figures 9–11). The central canal was surrounded by a prominent, continuous subependymal astrocytic layer in immediate apposition to the ependymal lining (Figure 9), with astrocytic processes occasionally extending between ependymocytes and reaching the central canal lumen (Figure 11).

Surrounding the central canal, there was gray matter containing scattered neuronal bodies within a neuropil populated by glial cells and blood vessels (Figures 8–11). Immunohistochemical analysis revealed neurons of varying sizes that were immunoreactive for NSE and PGP 9.5 (Figures 9–11). These neurons were interspersed among astrocytic processes that demonstrated positive immunoreactivity for GFAP.

White matter containing myelinated axons was also identified in the FTi (Figure 9). A continuous astrocytic layer (external glial limiting membrane) was present at the periphery, directly adjacent to the pia mater (Figures 9, 10). Blood vessels penetrating both gray and white matter were consistently ensheathed by perivascular astrocytic endfeet (Figure 9).

Within the dural sac, myelinated nerve fibers and spinal ganglia were identified within the periphery of the FTi, embedded in thin fascicles of connective tissue (Figures 7, 8). These connective tissue elements exhibited positive staining with Masson’s trichrome, confirming their collagenous composition.

**Figure 12:**
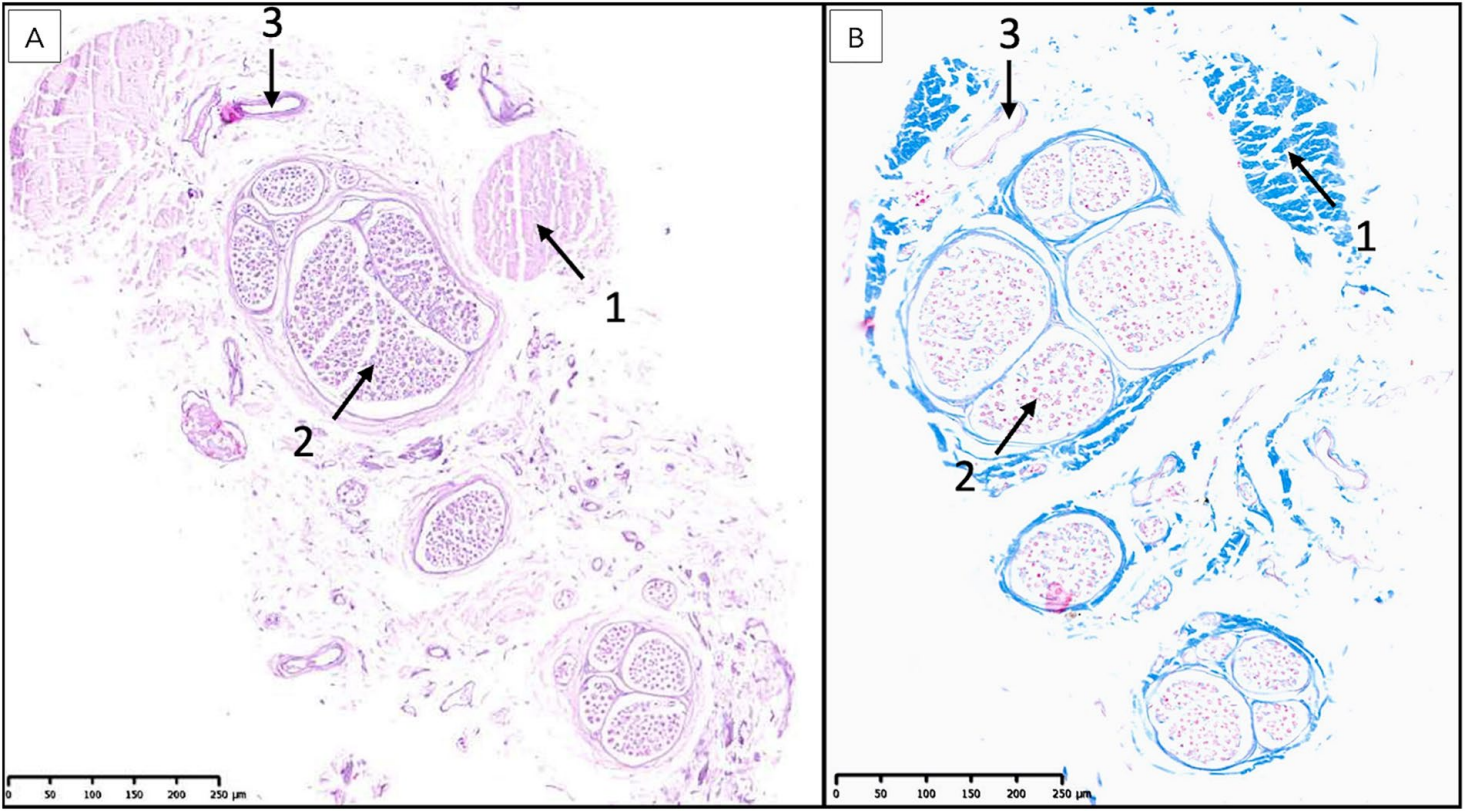
Histological examination of the most caudal portion of the filum terminale externum at the level of the first caudal vertebra. **(A)** Hematoxylin and eosin (H&E) staining reveals a predominance of bands of dense connective tissue (1), interspersed with isolated nerve fibers (2) and blood vessels (3). The absence of a central canal surrounded by nervous tissue and the marked increase in connective tissue are characteristic of the terminal region of the filum terminale. **(B)** Masson’s trichrome staining further highlights the extensive collagenous connective tissue (1, blue), with scattered nerve fibers (2) and blood vessels (3) embedded within the matrix, confirming the transition to a primarily fibrous structure at this level.

The above-described neural elements of the FT progressively diminished as it extended caudally. The number of neuronal cells decreased near the first sacral vertebra; however, it retained abundant astrocytic support and myelinated nerve fibers. At the level of the last sacral vertebra, the FTe became thinner, and the neuronal population diminished notably. An immature mass of mesenchymal cells producing collagen was observed adjacent to these neural remnants (Figures 10, 11). This connective tissue component thickened significantly at the caudal portion of the FT at Cd1 vertebra (Figure 12).

As observed at the level of the FTi, longitudinal sections of the FTe revealed an irregular central canal, attributable to abundant folds observed in transverse sections (Figure 11). Near the first caudal vertebra, ependymal cells could still be observed but were markedly diminished, as were the surrounding neural components, leaving predominantly connective tissue interspersed with occasional bundles of myelinated axons, likely representing residual nerve fibers (Figure 12). Notably, VVG staining for elastic tissue revealed a sparse distribution of elastic fibers within the collagen matrix, although these fibers were preserved in the elastic laminae of blood vessels.

## Discussion

The FT represents a neuroanatomical structure with a unique tissue configuration whose detailed characterization in healthy dogs has been notably absent from veterinary literature until now. This study addresses this critical knowledge gap by establishing baseline anatomical and histological parameters of this complex structure, which transitions from neural to predominantly connective tissue along its course. Such baseline data are essential for future comparative studies and for recognizing deviations that may underlie clinical conditions.

The FT has traditionally been regarded as a mere fibrous extension of the spinal cord; however, contemporary investigations from human medicine indicate its more complex nature with dual mechanical and potential neurofunctional properties. Biomechanical analyses (9) revealed that the FT exhibits elastic qualities, absorbing substantial strain under axial loading and thereby attenuating traction forces on the CM, in concert with the dentate ligaments. In parallel, histological examinations (33) demonstrated the presence of neuropil within the FT, confirming both astroglial and neuronal components. Further advancing this understanding, Gaddam et al. (3) established anatomical continuity between the FT and adjacent nerve roots, while subsequent characterization by Picart et al. (34) identified nerve fibers, ependymal cells, and even mechanoreceptors within this structure. Similarly, Klinge et al. (14) described Ruffini-like mechanoreceptors and nociceptive glioneural elements within the FT. Neurophysiological evidence has subsequently emerged through electrophysiological studies demonstrating that morphologically normal FT can generate electromyographic responses in paraspinal (14), lower limb and sphincter musculature comparable to those elicited by cauda equina motor nerve roots (17). Notably, this electrophysiological function is absent in pathological FT (17). Collectively, these findings substantiate that the FT functions not merely as a passive anchoring structure but as a sophisticated fibro-neural entity potentially involved in both mechanical load distribution and neural transmission processes (14).

Similarly to the human FT, our histological examination of neurologically normal canine specimens revealed a complex neural structure with preserved, albeit vestigial, spinal cord cytoarchitecture. The canine FT also exhibited an irregular central canal lined with ependymal cells, surrounded by a prominent subependymal astrocytic layer that showed strong GFAP immunoreactivity. Notably, we identified scattered neuronal bodies within the gray matter that were immunopositive for NSE and PGP 9.5, along with myelinated axons in the white matter. While Gaddam et al. (3) documented nerve bundles and cells positive for GFAP, synaptophysin, S100, and nestin in human FT specimens, our canine study utilized different neural markers to identify neuronal populations, and a direct immunohistochemical comparison is not possible. The presence of myelinated nerve fibers observed in human specimens was also evident in our canine tissue. Picart et al. (34) described axons and ependymal cells in the human FT with a proximal-to-distal gradient, which aligns with our observation of progressively diminishing neural elements as the canine FT extended caudally. These cross-species comparisons highlight conserved interspecies features, further supporting its functional significance beyond a simple fibrous anchor.

Building upon our findings, the comparative analysis of canine and human FT also reveals a significant difference with potential functional implications. Our VVG staining demonstrated that the canine FT contains a sparse distribution of elastic fibers within its collagen-rich matrix. This contrast with the human FT, which is characterized by abundant elastic and elaunin fibers interwoven with robust longitudinal Type I and transverse Type III collagen bundles (10). While both species share a collagen-dominant architecture, the interspecies variation in elastic fiber content likely influences tissue biomechanics. This also raises the hypothesis that the biomechanical properties of the FT and its susceptibility to pathologies like TCS may depend more on collagen organization and quantity than on elastic fiber abundance—a notion supported by pediatric histological data showing that control normal fila exhibit loose fibrous connective tissue (FCT), while TCS-affected fila consistently show abnormal dense FCT, even when elastic fibers are preserved (35). Furthermore, we observed a regional maturation pattern in the connective tissue of the canine FT, transitioning from cellular, collagen-producing mesenchymal tissue at the sacral level to a denser, more fibrous collagen matrix as it extends toward the first caudal vertebrae. This suggests that not only the composition but also the developmental remodeling of connective tissue may influence the mechanical properties of the FT, challenging conventional understanding and underscoring the need for further studies in the canine species. Future studies should ideally incorporate ultrastructural techniques, such as electron microscopy, to better characterize fiber types, organization, and orientation. They should also include juvenile animals to account for developmental variability and enable comparison with age-matched, pathologically affected counterparts. These approaches will be essential to further investigate the relationship between extracellular matrix composition and clinical manifestations, particularly in pathological conditions such as occult TCS.

Our findings provide histological evidence of a folded, ependyma-lined central canal within the FT, composed of multiple elongated cavities seen in longitudinal sections. Corresponding transverse sections confirmed these to be contiguous, irregularly contoured segments of a single central canal. This morphology closely resembles the embryologically derived ventriculus terminalis (36), which is a transient, ependyma-lined dilatation of the distal central canal in the CM. The irregular contours observed in ventriculus terminalis histology are paralleled in our observations, supporting a continuous developmental trajectory between the central canal of the spinal cord, ventriculus terminalis, and the central canal of the FT. These findings are well explained by the embryologic process of canalization and retrogressive differentiation. During canalization, vacuoles within the caudal cell mass coalesce into a single, ependyma-lined tube—the future central canal and ventriculus terminalis. Retrogressive differentiation then transforms the distal spinal cord into the FT. However, residual nests of ependymal cells often remain within the FT, and accessory lateral and dorsolateral canals, which may or may not communicate with the central canal, are seen in up to 35% of healthy adults (36). The folded, multi-locular but single canal structure we observed is consistent with this variability and supports the idea that the FT retains embryonic canal architecture.

A prominent and continuous layer of subependymal astrocytes was identified on histological examination of our canine FT specimens, with some astrocytic processes extending through the ependymal lining to contact the central canal. The morphology and positioning of these cells are reminiscent of tanycytes, which have been described in the third and fourth ventricles of mammals such as humans, rats, and rabbits (37, 38). Tanycytes are specialized glial cells thought to derive from radial glia, likely retaining certain embryological properties into adulthood. In the hypothalamus, they serve key functions in maintaining the interface between the CSF, blood, and neuronal tissue, mediating the transport of signaling molecules and contributing to neuroendocrine and metabolic regulation. Specific subtypes are capable of sensing nutrient levels and influence the release of hormones, including gonadotropin-releasing hormone and thyrotropin-releasing hormone (37). Additionally, they possess the ability to proliferate and generate new glial or neuronal cells after birth. Tanycytes in the human FT may be implicated in pathological conditions, such as the development of tanycytic ependymomas, emphasizing their active role—not merely as embryonic remnants but as functionally active cells with neurogenic potential that could contribute to tumorigenesis and FT pathology (39). Supporting this view, studies in rats and humans have identified the FT as a mitotically active region containing cells with immunocytochemical profiles similar to those found in known central nervous system stem cell niches. This suggests that the FT may serve as a source of ependymal or neural progenitor cells (40). These findings support the hypothesis that tanycyte-like cells identified in the canine FT may serve comparable structural, regulatory, or neurogenic roles, potentially contributing to cellular homeostasis, neurogenesis, or disease processes in this region.

The term FT is recognized by the *Nomina Anatomica Veterinaria* (NAV) (41), yet without clarification regarding its anatomical subdivisions. Similarly, current veterinary literature lacks a detailed description of its structural organization (7). As confirmed by our study, the anatomy of the human FT closely resembles that of the canine species. The most consistent accounts in human scientific literature describe two distinct segments: a cranial portion located within the dural sac (FTi), and a caudal portion (FTe), enclosed solely by a thick caudal extension of the inner dural layer, also referred to as the dural filament or *filum durae matris spinalis* (24). The absence of standardized terminology and anatomical precision may contribute to inconsistencies in diagnosis, clinical reporting, and surgical planning. Importantly, this segmentation is not merely of academic interest—it may have direct clinical relevance, particularly in the context of occult TCS, where pathological changes can involve either the entire FT or be confined to one of its segments. Precise identification of the affected portion is critical, as it directly informs the surgical approach required to relieve pathological tension. Although the terms FTi and FTe are not currently recognized by the NAV, they potentially possess clinical and neurosurgical value. We therefore propose their continued use and formal adoption in veterinary terminology to enhance anatomical accuracy, facilitate interdisciplinary communication, and support appropriate surgical management.

Recognition of anatomical landmarks in relation to the FT is critical for neurosurgical approaches aimed at accurately identifying and dissecting this structure. In our study, the origin of the cranial portion of the FT, the FTi, was located just caudal to the apex of the CM which was consistently identified within the region of L6 vertebra. The DS terminated at mid-sacrum, and the caudal portion of the FT, the FTe, extended caudally to terminate consistently on the *ligamentum flavum* between the first and second caudal vertebrae. This attachment—known in human anatomy as the coccygeal ligament (42)—anchors most frequently to the first coccygeal vertebra in nearly 70% of cadaveric specimens, typically via a single filamentous strand (61%) or, less often, multiple fine strands (39%). Macroscopically, the distal FTe presents a flattened profile in 64% of cases and is largely immobile in 60%. Notably, it is also enveloped by a previously undescribed distal coccygeal venous plexus at its insertion site (43). While our findings were consistent across all cadaveric specimens examined, it is important to acknowledge that the termination point of the CM—and thus the origin of the FT—may vary among dogs depending on body size. One study reported the CM termination ranging from cranial L6 to the cranial sacrum, while the DS termination, which appeared independent of body size, varied from cranial L7 to the caudal sacrum (44). It should be noted that our study did not aim to characterize breed- or size-related anatomical variation of the FT, and the lack of significant anatomical variability observed in our cohort does not reflect the broader canine population. Consequently, neurosurgical planning should be guided by careful assessment of individual anatomical configurations as visualized on preoperative MRI studies.

This study presents several limitations that warrant consideration. The small sample size of eight cadaveric dogs, representing a limited range of breeds and body sizes, constrains the generalizability of the findings and precludes a robust analysis of breed-or weight-related anatomical variations. Furthermore, the exclusive use of adult dogs does not account for potential age-related changes in the morphology or composition of the filum terminale. Although most specimens were processed promptly postmortem, the possibility of tissue degradation affecting fine histological features cannot be entirely excluded. Additionally, while conventional histological and immunohistochemical techniques were employed, the absence of advanced modalities such as electron microscopy or confocal immunofluorescence restricted the resolution of ultrastructural and three-dimensional cellular arrangements, particularly for small neural and vascular components. Lastly, functional studies were beyond the scope of this investigation, limiting the correlation of morphological findings with biomechanical or neurophysiological roles.

## Conclusion

This study provides the first comprehensive anatomical and histological characterization of the FT in neurologically healthy adult dogs, revealing it as a structurally complex and developmentally derived continuation of the spinal cord. The presence of residual neural elements, glial support, and connective tissue maturation patterns suggests that the FT serves more than a passive anchoring function. These findings establish a foundational reference for future investigations into pathologies involving the FT such as TCS. Expanding this research to include broader canine populations and integrating morphometric, ultrastructural, biomechanical and molecular analyses will be essential for advancing our understanding of FT physiology and pathology in veterinary medicine.

## Data Availability

The original contributions presented in the study are included in the article/Supplementary material, further inquiries can be directed to the corresponding author.
