# Supplementary material for: Anatomical and histological characterization of the filum terminale in dogs
Source: Front Vet Sci. 2025 Jul 24;12:1650893. doi: 10.3389/fvets.2025.1650893 (PMC12330388; doi:10.3389/fvets.2025.1650893)
Supplement: Supplementary file 1 [file Data_Sheet_1.pdf]

Supplementary Table 1. Breed, sex, and body weight of eight adult canine cadavers used for anatomical and histological study of the filum terminale.

**Case No.**	**Breed**	**Sex**	**Weight** (kg)
**1**	Beagle	female	11.3
**2**	Labrador Retriever	male	34
**3**	Golden Retriever	male	31.8
**4**	German Shepherd	male	36.3
**5**	Border Collie	female	20.4
**6**	mixed breed	female	18.1
**7**	mixed breed	female	20.4
**8**	mixed breed	female	13.6
